# Inhibition of leucine-rich repeats and calponin homology domain containing 1 accelerates microglia-mediated neuroinflammation in a rat traumatic spinal cord injury model

**DOI:** 10.1186/s12974-020-01884-4

**Published:** 2020-07-06

**Authors:** Wen-Kai Chen, Lin-Juan Feng, Qiao-Dan Liu, Qing-Feng Ke, Pei-Ya Cai, Pei-Ru Zhang, Li-Quan Cai, Nian-Lai Huang, Wen-Ping Lin

**Affiliations:** 1 Department of Orthopedic Surgery, the Second Affiliated Hospital, Fujian Medical University, Quanzhou, 362000 China; 2 Department of Neurology, Fujian Medical University Union Hospital, Fuzhou, 350001 China; 3 Department of Head and Neck Oncology, The Cancer Center of The Fifth Affiliated Hospital of Sun Yat-Sen University, Zhuhai, 519001 China; 4 Department of Obstetrics and Gynecology, the Second Affiliated Hospital, Fujian Medical University, Quanzhou, 362000 China; 5 Department of Spine Surgery, Shenzhen Pingle Orthopedic Hospital, Shenzhen, 518001 China

**Keywords:** Leucine-rich repeats and calponin homology domain containing 1, Spinal cord injury, Microglia, Neuroinflammation, Mitogen-activated protein kinase

## Abstract

**Background:**

Spinal cord injury (SCI) triggers the primary mechanical injury and secondary inflammation-mediated injury. Neuroinflammation-mediated insult causes secondary and extensive neurological damage after SCI. Microglia play a pivotal role in the initiation and progression of post-SCI neuroinflammation.

**Methods:**

To elucidate the significance of LRCH1 to microglial functions, we applied lentivirus-induced LRCH1 knockdown in primary microglia culture and tested the role of LRCH1 in microglia-mediated inflammatory reaction both in vitro and in a rat SCI model.

**Results:**

We found that LRCH1 was downregulated in microglia after traumatic SCI. LRCH1 knockdown increased the production of pro-inflammatory cytokines such as IL-1β, TNF-α, and IL-6 after in vitro priming with lipopolysaccharide and adenosine triphosphate. Furthermore, LRCH1 knockdown promoted the priming-induced microglial polarization towards the pro-inflammatory inducible nitric oxide synthase (iNOS)-expressing microglia. LRCH1 knockdown also enhanced microglia-mediated N27 neuron death after priming. Further analysis revealed that LRCH1 knockdown increased priming-induced activation of p38 mitogen-activated protein kinase (MAPK) and Erk1/2 signaling, which are crucial to the inflammatory response of microglia. When LRCH1-knockdown microglia were adoptively injected into rat spinal cords, they enhanced post-SCI production of pro-inflammatory cytokines, increased SCI-induced recruitment of leukocytes, aggravated SCI-induced tissue damage and neuronal death, and worsened the locomotor function.

**Conclusion:**

Our study reveals for the first time that LRCH1 serves as a negative regulator of microglia-mediated neuroinflammation after SCI and provides clues for developing novel therapeutic approaches against SCI.

## Background

Spinal cord injury (SCI) triggers the primary mechanical injury and secondary inflammation-mediated injury [1]. The mechanical trauma of the spinal cord tissue initiates the primary injury, while the secondary neuroinflammatory reactions, which mediate additional and extensive neurological injury, take place following the primary injury. Controlling the detrimental acute neuroinflammation could be a therapeutic strategy to confine the injury and promote functional recovery.

Microglia play a pivotal role in the post-SCI secondary injury. During the past decade, various aspects of microglial heterogeneities within distinct regions of the central nervous system and the potential intrinsic and extrinsic factors regulating the heterogeneities have been unveiled [2]. Putative microglial subtypes have been proposed based on self-renewal and turnover rates, surface molecule expression, and transcriptome profiles under steady-state or pathological conditions [3]. To date, several polarized microglia populations have been described: classic activation (M1), alternative activation (M2a), alternative type II activation (M2b), and acquired deactivation (M2c) [4]. After SCI, resident microglia are distributed on the edge of the lesion [5]. Previous studies showed that microglia clear damaged and degenerate tissues [6], while depletion of microglia after SCI reduces neuronal and oligodendrocyte survival and impairs locomotor recovery [7]. It seems the overall effect of microglia is beneficial to spinal cord recovery. However, in the acute phase of SCI, activated microglia can produce pro-inflammatory cytokines such as TNF-α, IL-1β, and IL-6 which are neurotoxic [8, 9]. Therefore, carefully tuning the microglia function to minimize their detrimental effect and boast their neuroprotective effect is vital to promote neurological recovery.

Leucine-rich repeats and calponin homology domain containing 1 (LRCH1) is a relatively novel gene that encodes a protein with a leucine-rich repeat and a calponin homology domain. The significance of LRCH1 protein remains a mystery. Previous studies suggest that a genetic variant in LRCH1 is associated with knee osteoarthritis [10, 11]. A recent study indicates that LRCH1 act to restrain PKCα-DOCK8-Cdc42 module-mediated T cell migration in experimental autoimmune encephalomyelitis, through competing with Cdc42 for interaction with DOCK8 [11]. In addition, LRCH1 seems to be associated with tumors. The *Lrch1* gene loci are among the most recurrently aberrant regions in prostate cancer patients [12]. LRCH1_E > K_ mutation is found in melanoma, making LRCH1 a neoantigen recognized by CD8+ T cells [13]. LRCH1 is overexpressed in colorectal carcinoma [14]. However, the functions of LRCH1 in tumors are yet to be determined, although LRCH1 transgenic mice and LRCH1 knockout mice have been recently generated.

In our unpublished pilot RNA sequencing study, LRCH1 was found to be downregulated in microglia in the acute stage of SCI. In the current research, we found that LRCH1 was downregulated in microglia after traumatic SCI. To elucidate the significance of LRCH1 in microglial functions, we applied lentivirus-induced LRCH1 knockdown in primary microglia. Our study reveals for the first time that LRCH1 serves as a negative regulator of microglia-mediated neuroinflammation after SCI, and provides clues for developing novel therapeutic approaches against SCI.

## Materials and methods

### Rat SCI model

The animal study was approved by the Animal Care and Use Committee of the Second Affiliated Hospital of Fujian Medical University and Shenzhen Pingle Orthopedic Hospital. The surgical procedures were conducted in compliance with the institutional guidelines for laboratory animal usage in neuroscience and behavioral research. Male Sprague-Dawley rats (10 weeks old, 250~300 g) were purchased from Beijing Vital River Laboratory Animal Technology Co., Ltd. and were housed in the pathogen-free condition.

The SCI model was established by the following steps. Rats were anesthetized via inhaling 3% of isoflurane at the flow rate of 1 L/min. Midline skin incisions were cut, and the T12 spinous processes were exposed. A laminectomy was performed at T12. The compression was conducted by placing the base of a compression platform (area 2 × 5 mm^2^) onto the exposed spinal cord. A 50-g weight was then placed steadily to the platform for 5 min. The platform was then removed, and the muscles and skins were sutured. Rats were transferred to the cages after they regained the righting reflex. The urinary retention was relieved by twice-daily bladder expressions. The sham-operated rats received every surgical step except for the spinal cord compression.

### Immune cell enrichment from the spinal cords

Rats were anesthetized by inhalation of 3% isoflurane. Each rat was transcardially perfused with 200 ml of ice-cold phosphate-buffered saline (PBS). The spinal cord was taken, minced into approximately 1-mm^3^ pieces, and treated with RPMI1640 supplemented with 2 mg/ml collagenase IV (Thermo Fisher Scientific), 200 U/ml DNase I (Sigma-Aldrich), 10% fetal bovine serum (FBS), and 2.0 mM CaCl_2_ for 30 min at 37 °C in an incubator. Digested spinal cord tissues were then filtered through 70-μm cell strainers and overlaid onto 20% Percoll (GE Healthcare), followed by centrifugation at 250*g* for 10 min. The cell pellet was resuspended in PBS or culture medium before further experiments. In some experiments, the spinal cords of 3 to 5 rats were pooled to collect enough cells.

### Flow cytometry and cell sorting

The following fluorophore-conjugated antibodies were purchased from Biolegend: APC anti-granulocyte (RP-1), PE anti-CD45 (OX-1), APC anti-TCRαβ (R73), PE anti-TCRγδ (V65), and PE-Cy7 anti-CD11b (OX-42). Polyclonal APC anti-Arginase-1 antibody (IC5868A) was purchased from Novus Biologicals. Unconjugated polyclonal anti-inducible nitric oxide synthase (iNOS) antibody (ab15323) was purchased from Abcam. For cell surface marker staining, cells were stained with 2 μg/ml each antibody on ice for 30 min. Dead cells were excluded by staining with 1 μg/ml propidium iodide (PI; ebioscience). For intracellular staining, cells were fixed with 4% paraformaldehyde for 15 min and permeabilized with 90% ice-cold methanol for 30 min, followed by incubation with 1:100 diluted anti-Arginase-1 antibody and 10 μg/ml anti-iNOS antibody for 1 h at room temperature. After three washes with PBS, cells were incubated with 1 μg/ml Alexa Fluor® 594 goat anti-rabbit IgG (ab150080, Abcam) for 30 min. For apoptosis assay, cells were stained with PI and Annexin V following the instructions of the APC Annexin V Apoptosis Detection Kit with PI (Biolegend). Cells were then washed with PBS twice and loaded onto either a BD LSR-II flow cytometer (BD Biosciences) for analysis or a BD FACSAria II sorter (BD Biosciences) for sorting.

### Quantitative RT-PCR (qRT-PCR)

Cellular RNAs were purified using the ARCTURUS PicoPure RNA Isolation Kit (Thermo Fisher Scientific). Tissue RNAs were extracted using the Trizol reagent (Thermo Fisher Scientific) following the vendor’s manual. SuperScript® III First-Strand Synthesis Kit (Thermo Fisher Scientific) was used to prepare cDNA. On a 7300 qPCR thermocycler (Invitrogen), quantitative PCR was achieved using Fast SYBR®Green Master Mix (Thermo Fisher Scientific) at the following steps: 50 °C for 2 min, then 94 °C for 10 min, and then 40 cycles of 30 s at 94 °C and 1 min at 60 °C. The data were analyzed using the 2^-ΔΔCt^ method. The primers are listed in Supplementary Table 1.

### Primary rat microglia culture

Primary microglia were obtained by isolation from mixed glial cell cultures of 1-day-old neonatal rat spinal cords, according to previous publications [15–17]. Briefly, after removing the meninges and blood vessels, the spinal cords were minced and incubated in 0.25% trypsin-EDTA (Invitrogen) for 30 min at 37 °C while shaking at 50 rpm on an orbital shaker. The tissue was then mechanically dissociated in DMEM (Thermo Fisher Scientific) supplemented with 10% FBS and 200 U/ml DNase I until no tissue clump was seen. The released spinal cord cells were passed through a 70-μm cell strainer and washed with DMEM once. The cells were then cultured at 5 × 10^6^ cells/ml in DMEM supplemented with 10% FBS, and the medium was replaced every 3 days. Two weeks later, the culture was mildly trypsinized with 1:4 diluted 0.25% trypsin-EDTA for 20 min at room temperature. The floating cells were carefully aspirated, and microglia which remained attaching to the bottom were cultured in the same culture medium before further experiments.

### Lentiviral packaging and transduction

Lrch1-set siRNA/shRNA/RNAi Lentivector (i050632) and the corresponding control lentivector piLenti-siRNA-GFP were purchased from abmgood Inc. The packaging was conducted using the Ecotropic Lentiviral Packaging System (VPK-205, Cell Biolabs, Inc.) according to the vendor’s protocol. The lentiviruses were purified with Lenti-X™ Maxi Purification Kit (Clontech). The viral titer was determined by Neuronbiotech Company. The lentivirus containing the LRCH1 shRNA sequence was termed “LL” while the control virus was termed “LC.”

Primary microglia were cultured at the density of 2 × 10^6^ cells/ml in DMEM supplemented with 10% FBS, 4 mM l-glutamine, and 50 μg/ml penicillin/streptomycin, and in the presence of 6 μg/ml polybrene (Thermo Fisher Scientific) in 48-well plates. Lentiviral particles were added into microglia culture at the MOI of 20 and incubated overnight. The next morning, the medium was then replaced with fresh medium. Cells were incubated in fresh medium for 2 days, followed by incubation with 2 μg/ml puromycin (Sigma-Aldrich) for 4 days.

### In vitro treatment of microglia

1 × 10^6^/ml lentivirus-infected microglia were primed with 20 ng/ml lipopolysaccharide (LPS; Sigma-Aldrich) for 6 h (or 1 h if specified) followed by treatment with 5 mM adenosine triphosphate (ATP) for additional 1 h.

The N27 rat dopaminergic neural cell line was purchased from Sigma-Aldrich. N27 cells were cultured in RPMI 1640 medium supplemented with 10% FBS in a humidified atmosphere of 5% CO_2_ at 37 °C. N27 cells were differentiated with 2 mM dibutyryl cAMP (D0260, Sigma-Aldrich) for 5 days and then used for the experiment described below. To test microglia-mediate neuron death, 5 × 10^4^ N27 cells were cultured in each well of a Transwell 96-well plate (Corning). 1 × 10^5^ unprimed or primed primary microglia were seeded into the Transwell 0.4-μm pore membrane insert. N27 cells and microglia were then co-cultured without direct contact for 24 h. N27 cells were then lifted by 5-min trypsinization at 37 °C with 0.25% trypsin-EDTA. N27 cell apoptosis and necrosis were then determined using the APC Annexin V Apoptosis Detection Kit with PI (Biolegend).

Primary rat spinal cord neurons were purchased from Guangzhou Ubigene Biosciences and cultured in Neurobasal-A medium supplemented with the B27 supplement. 1 × 10^4^ neurons were seeded in each well of a Transwell 96-well plate and were labeled with 5 μM carboxyfluorescein succinimidyl ester (CFSE; Thermo Fisher) following the vendor’s instructions. 5 × 10^4^ unprimed or primed primary microglia were then seeded into the Transwell 0.4-μm pore membrane insert. Neurons and microglia were co-cultured without direct contact for 24 h. After that, the insert was removed, and neurons were rinsed with 100 μl of PBS twice before CFSE intensity was read on a Tecan Infinite 200 PRO plate reader.

### Elisa

The culture supernatants were collected and centrifuged at 250 g for 5 min before storage at − 80 °C. Microglia were lysed using the denaturing M-PER Mammalian Protein Extraction Reagent (78503, Thermo Fisher Scientific) supplemented with the protease inhibitor cocktail (S8820, Sigma-Aldrich) for 5 min on ice. The cell lysates were then centrifuged at 10000*g* for 5 min before storage at − 80 °C. The indicated cytokines were measured with the Rat IL-6 ELISA Set (550319, BD Biosciences), Rat TNF ELISA Set (558535, BD Biosciences), and Rat IL-1 beta ELISA Kit (ab100768, Abcam), respectively.

### Immunoblotting

Rabbit anti-LRCH1 antibody (bs-9327R) was purchased from Beijing Bioss Antibodies Inc. The following antibodies were purchased from Cell Signaling Technology: beta-actin antibody (3700), phospho-p38 mitogen-activated protein kinase (MAPK) (Thr180/Tyr182) antibody (9216), phospho-SAPK/Jun N-terminal kinase (JNK) (Thr183/Tyr185) antibody (9255), phospho-p44/42 MAPK (Erk1/2) (Thr202/Tyr204) antibody (9101), p44/42 MAPK (Erk1/2) antibody (4695), p38 MAPK antibody (8690), and SAPK/JNK Antibody (9252).

### Adoptive transfer of microglia

The adoptive transfer procedure was conducted based on established approaches with several modifications [18–20]. Briefly, immediately before SCI, 1 × 10^6^ lentivirus-infected microglia in 5 μl of 0.9% NaCl solution was injected into the SCI area (epicenter) at a depth of 1 mm at the rate of 0.2 μl/min, using a 5-μl micro-syringe with a 33-gauge Hamilton needle. Rats in the vehicle group received the same amount of saline. After injection, the injectors were removed and the compression of the spinal cord was conducted to induce SCI as described above. After that, muscles and skins were sutured in separate layers.

### Hematoxylin and eosin (H&E) staining and immunofluorescent staining

Rats were transcardially perfused with ice-cold PBS followed by cold 4% paraformaldehyde (PFA). Spinal cords were then fixed in 4% PFA for 16 h. The next day, spinal cords were immersed in 30% sucrose-PBS for 3 days. Spinal cords were then embedded in paraffin. Five-micron-thick cross-sections were prepared and stained following the standard H&E staining protocol.

To detect the transferred microglia and endogenous microglia, the spinal cord sections were stained with the GFP antibody (1:1000, Abcam ab13970) and Iba1 antibody (10 μg/ml, Abcam ab5076) at 4 °C overnight. Alexa Fluor® 488-conjugated donkey anti-rabbit IgG (5 μg/ml, Abcam ab150073) and Alexa Fluor® 594-conjugated donkey anti-goat IgG (5 μg/ml, Abcam ab150132) were used as secondary antibodies to incubate the sections for 1 h at room temperature.

### Terminal deoxynucleotidyl transferase dUTP nick end labeling (TUNEL)

Double staining of TUNEL and the neuronal marker NeuN was performed to discern apoptotic neurons. Briefly, the spinal cord sections were stained with 2 μg/ml anti-NeuN antibody (Abcam ab128886) and then the Alexa Fluor 647-conjugated goat anti-rabbit IgG (Abcam ab150083). The sections were then subjected to staining with the TUNEL Assay Kit-BrdU-Red (Abcam ab66110) following the manufacturer’s manual. The percentage of TUNEL^+^NeuN^+^ cells in NeuN^+^ cells was calculated.

### Neurologic evaluation

The hindlimb locomotor function was evaluated before and 1, 3, 7, 14, and 21 days after SCI using the BBB locomotor test developed by Basso et al. [21]. The hindlimb movements during locomotion were quantified using a scale ranging from 0 to 21. The rats were observed for 5 min at each time point by two observers who were blinded to the study.

### Statistical analysis

Experiments were independently conducted three times unless specified, with 6 to 10 different samples in each group. Data were shown as mean ± standard deviation and were analyzed by GraphPad Prism 7.0. Student’s *t* test or one-way ANOVA was used to compare the mean values among the groups. *P* value < 0.05 was considered significant.

## Results

### LRCH1 is downregulated in post-SCI microglia

**Fig. 1:**
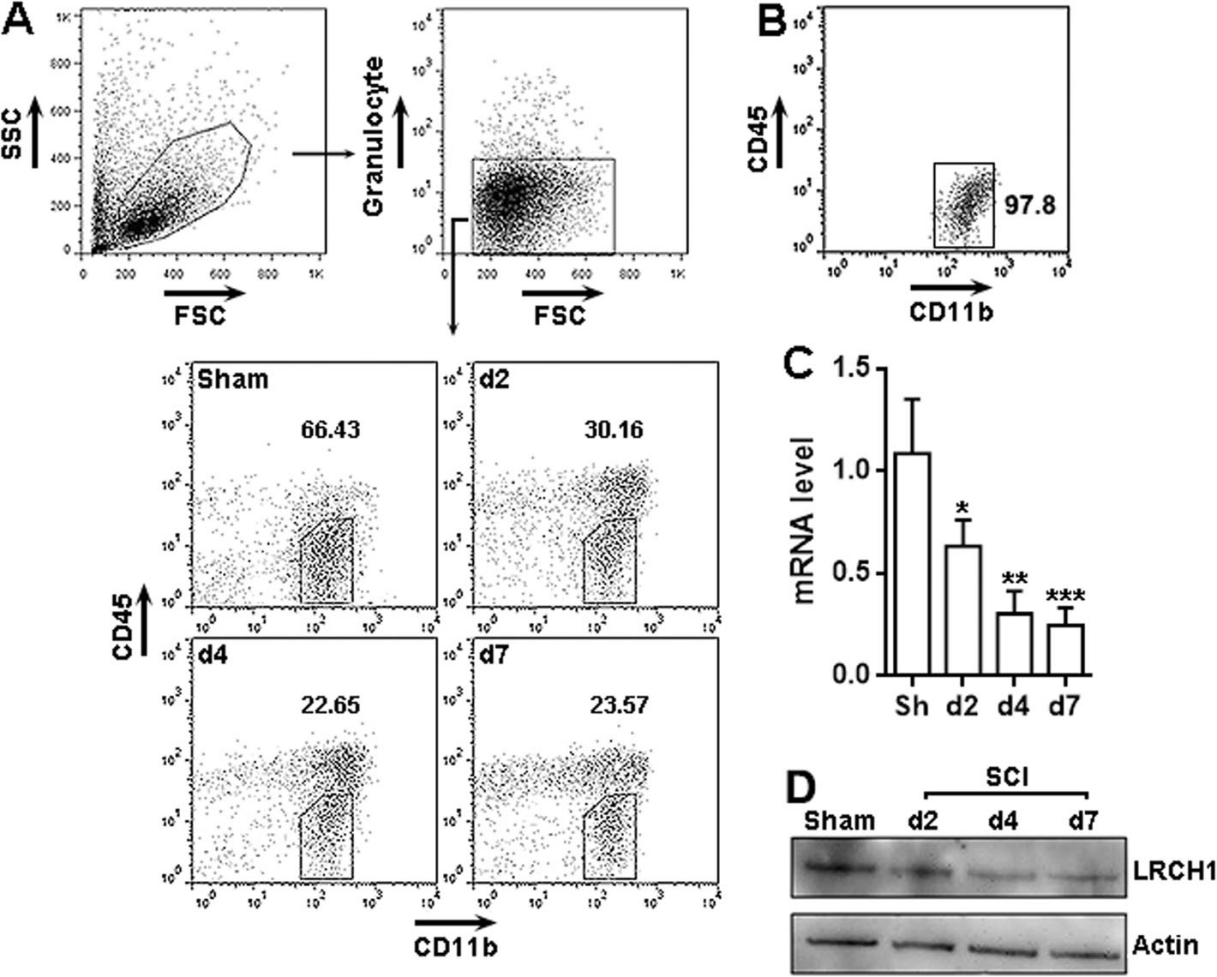
LRCH1 expression is reduced in post-SCI microglia. **a** The gating and sorting of spinal cord microglia using flow cytometry. Granulocyte^-^ cells were first gated from the whole enriched leukocytes, and CD45^low^CD11b^+^ microglia were then recognized and sorted. d2~d7, day 2 to day 7 after SCI. **b** The purity of sorted microglia. Sorted microglia were re-tested for the expression of CD45 and CD11b. **c** LRCH1 mRNA levels in sorted microglia at indicated time points after SCI. Sh, sham control. *n* = 5 in 3 independent experiments. **d** LRCH1 protein levels in sorted microglia. The data represents two independent experiments. *p < 0.05; **p < 0.01; ***p < 0.001

To determine LRCH1 expression in microglia, we first enriched leukocytes from the spinal cords of sham-operated rats and SCI rats (Fig. 1a). Granulocyte^-^CD45^low^CD11b^+^ were then sorted from the whole leukocyte population using flow cytometry (Fig. 1 a and b). The LRCH1 mRNA quantity in sorted microglia was determined by qRT-PCR. We found that LRCH1 mRNA was decreased in microglia in a time-dependent manner after SCI (Fig. 1c). LRCH1 protein was abundant in normal microglia and underwent similar decreases on day 2, day 4, and day 7 after SCI (Fig. 1d).

### LRCH1 knockdown elevates the production of pro-inflammatory cytokines

**Fig. 2:**
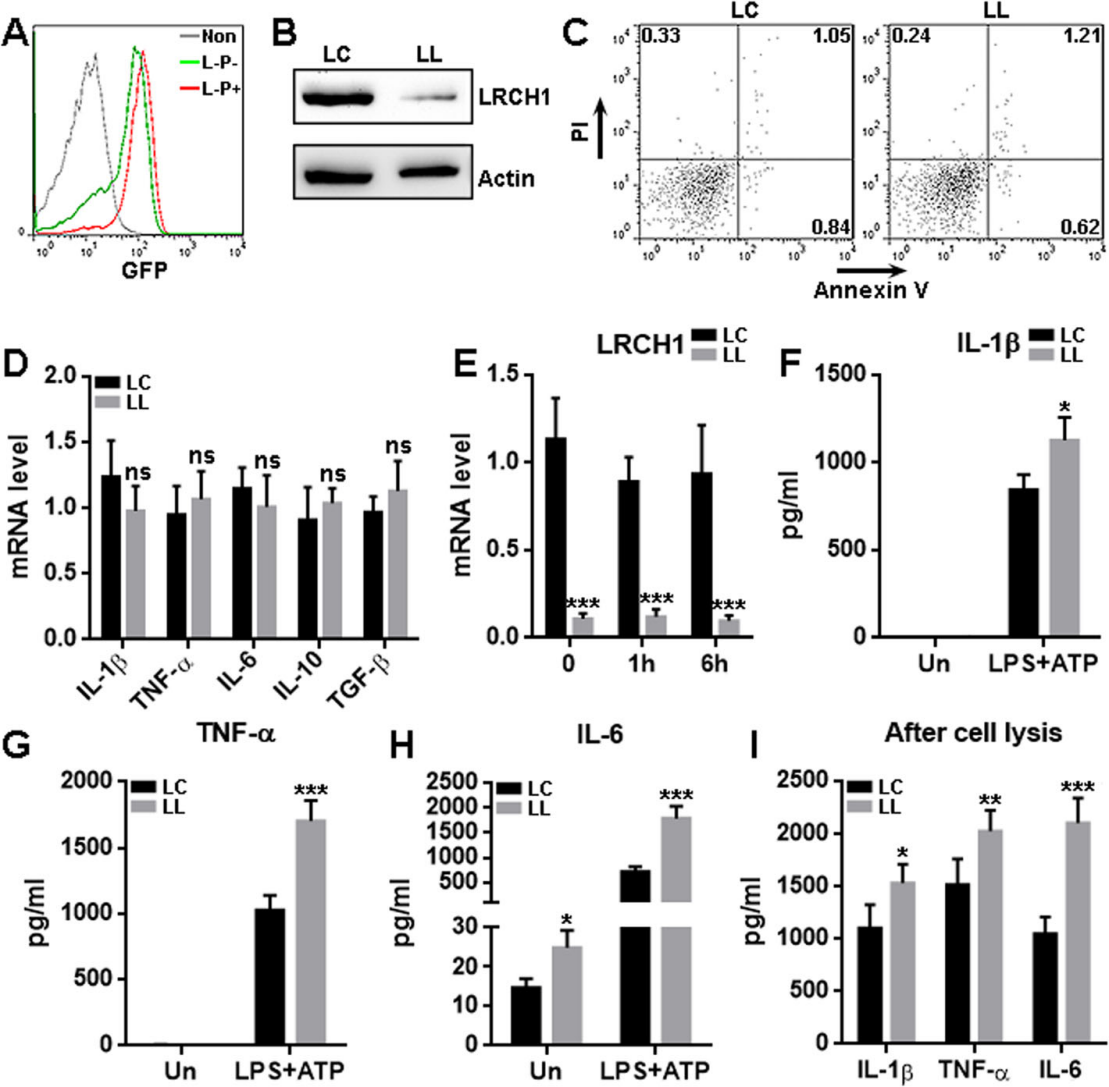
LRCH1 knockdown enhances the expression of pro-inflammatory cytokines in primed microglia. **a** GFP expression in lentivirus-infected microglia. Non, non-infected microglia. L-P−, lentiviral infection without puromycin selection. L-P+, lentiviral infection followed by puromycin selection. Note that only the cells infected with control lentivirus are shown here. The data are representative of two independent experiments. **b** LRCH1 protein in microglia after lentiviral infection and puromycin selection. LC, cells infected with control lentivirus. LL, cells infected with LRCH1 shRNA-encoding lentivirus. The data are representative of two independent experiments. **c** Microglia apoptosis and necrosis after lentiviral infection. The data are representative of three independent experiments. **d** mRNA levels of indicated cytokines in microglia after lentiviral infection. ns, non-significant. *n* = 3 in 3 independent experiments. **e** LRCH1 mRNA in microglia before (0) or after (1 or 6 h) priming with LPS + ATP. *n* = 4 in 3 independent experiments. **f–h** Concentrations of indicated cytokines in the supernatants of lentivirus-infected microglia culture after 6 h priming. Un, no priming. LPS + ATP, priming. *n* = 6 in 3 independent experiments. **i** Concentrations of indicated cytokines in the supernatant-lysate mixtures of lentivirus-infected microglia culture. *n* = 5 in 3 independent experiments. *p < 0.05; **p < 0.01; ***p < 0.001 in comparison to the “LC” group under the same conditions

One of the crucial roles of post-SCI microglia is to produce pro-inflammatory cytokines in the acute stage [5]. To ascertain whether LRCH1 is involved in microglia-mediated neuroinflammation, we infected primary rat spinal cord microglia with a lentivirus that expresses both LRCH1 shRNA and GFP, followed by puromycin selection. The infection efficiency reached approximately 90% after puromycin selection, as indicated by the proportion of GFP^+^ microglia on day 6 after infection (Fig. 2a and Supplementary figure 1). As shown in Fig. 2b, LRCH1 protein was drastically reduced in microglia infected with shRNA-encoding lentivirus (hereinafter LL), in comparison to microglia infected with the control lentivirus (hereinafter LC). LRCH1 knockdown had no remarkable impact on microglia survival or cytokine transcription under the in vitro normal state (Fig. 2 c and d). The lentiviral infection itself did not alter the expression of LRCH1 and these cytokines (Supplementary figure 2). These microglia were then primed with LPS plus ATP into a pro-inflammatory state. The priming did not alter LRCH1 transcription in either LC-infected or LL-infected microglia (Fig. 2e). The analysis of cytokines in the culture supernatants revealed that without priming, LL-infected microglia and LC-infected microglia secreted comparable traces of IL-1β and TNF-α (Fig. 2 f and g). After priming, both groups had dramatically high levels of IL-1β and TNF-α in the supernatants, whereas LL-infected microglia produced even more IL-1β and TNF-α than LC-infected microglia (Fig. 2 f and g). Concerning supernatant IL-6, no matter the priming was conducted or not, LL-infected microglia always secreted more IL-6 than LC-infected microglia (Fig. 2h). To investigate whether the above changes of supernatant cytokines were attributed to an overall downregulation of pro-inflammatory cytokine expression or deficient cytokine exocytosis, the microglia were lysed in a non-denaturing lysis buffer, and the lysates were mixed with the supernatants. The cytokines, including both mature and immature forms, were determined in the lysate-supernatant mixtures. It turned out that LL-infected microglia produced significantly more pro-inflammatory cytokines than LC-infected microglia (Fig. 2i).

### LRCH1 knockdown facilitates microglial polarization to the pro-inflammatory state in vitro

**Fig. 3:**
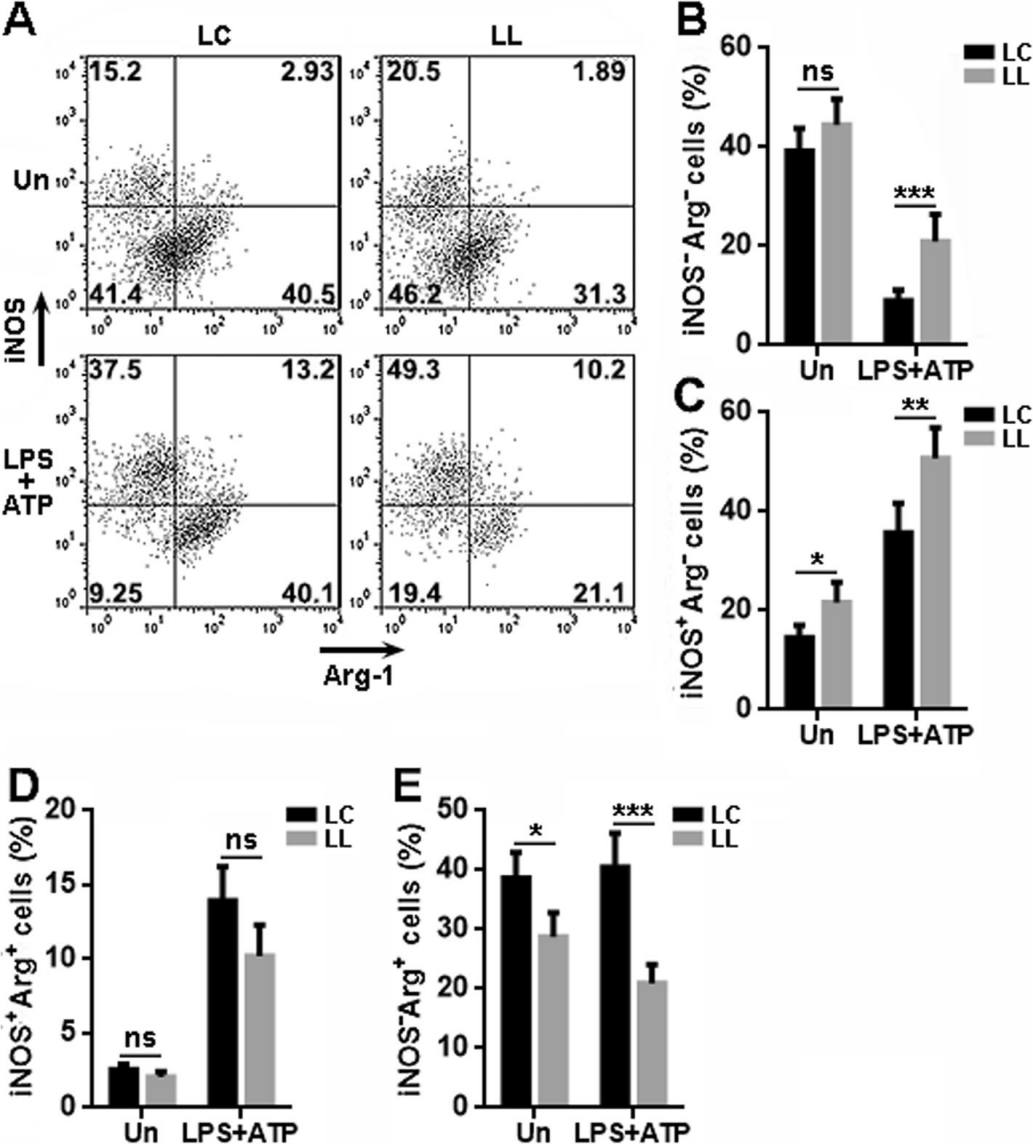
LRCH1 knockdown facilitates the microglial polarization to the pro-inflammatory state. **a** Representative dot plots showing the expression of iNOS and Arginase-1 in lentivirus-infected microglia after 6 h priming. **b**–**e** Statistics for the frequencies of iNOS^-^Arginase-1^-^ microglia (**b**), iNOS^+^Arginase-1^-^ microglia (**c**), iNOS^+^Arginase-1^+^ microglia (**d**), and iNOS^-^Arginase-1^+^ microglia (**e**). *n* = 8 in 4 independent experiments. ns, non-significant. *p < 0.05; **p < 0.01; ***p < 0.001

To further understand the significance of LRCH1, we analyzed the microglial polarization towards pro-inflammatory or anti-inflammatory type by detecting the classical activation marker iNOS and alternative activation marker Arginase-1 (Fig. 3a). Without priming, about 40% of either LC-infected microglia or LL-infected microglia were iNOS^-^Arginase-1^-^, while the priming decreased the frequency of this population in both LC-infected microglia and LL-infected microglia. However, primed LL-infected microglia still had higher iNOS^-^Arginase-1^-^ cells than primed LC-infected microglia (Fig. 3b). Unprimed LL-infected microglia had more iNOS^+^Arginase-1^-^ cells than unprimed LC-infected microglia, and this difference became bigger after LC-infected microglia and LL-infected microglia were primed (Fig. 3c). The frequency of iNOS^+^Arginase-1^+^ cells was comparable in LC-infected microglia and LL-infected microglia in the presence or absence of priming (Fig. 3d). Either before or after priming, there were fewer iNOS^-^Arginase-1^+^ cells in LL-infected microglia (Fig. 3e). Taken together, LL-infected microglia had less Arginase-1^+^ cells and more iNOS^+^ cells, suggesting that LRCH1 knockdown favors the conversion of microglia to the pro-inflammatory state.

### LRCH1 knockdown enhances microglia-mediated neuronal death

**Fig. 4:**
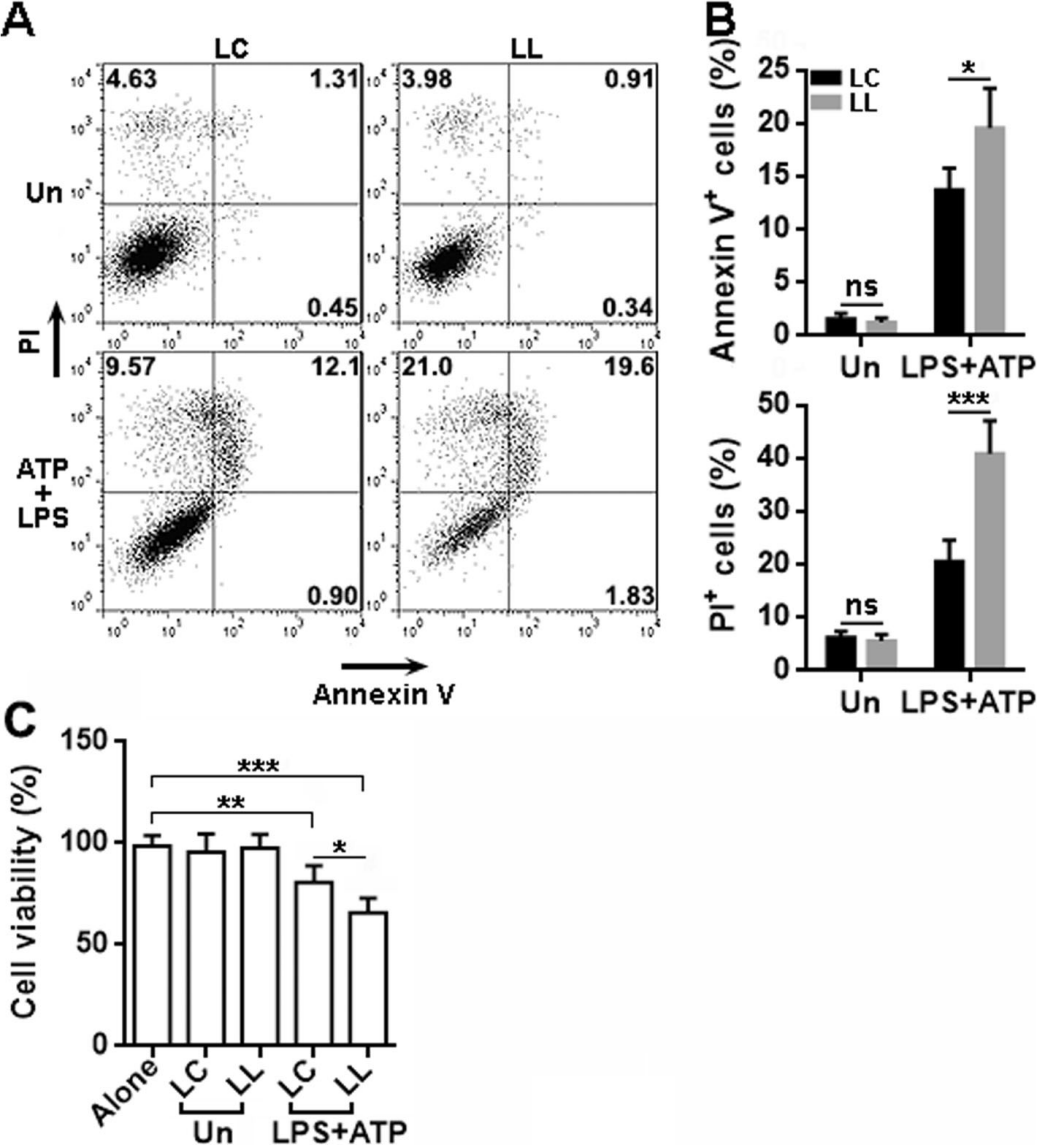
LRCH1 knockdown increases microglia-mediated N27 cell death. **a** Representative dot plots showing the apoptosis and necrosis of N27 cells after co-culture with primed lentivirus-infected microglia in Transwell plates. LC, N27 cells cultured with microglia infected with control lentivirus. LL, N27 cells cultured with microglia infected with LRCH1 shRNA-encoding lentivirus. Un, unprimed microglia. LPS+ATP, primed microglia. **b** Statistics for the frequencies of Annexin V^+^ cells and PI^+^ cells. Note that PI^+^ cells include PI^+^Annexin V^+^ cells and PI^+^Annexin V^-^ cells. *n* = 6 in 3 independent experiments. ns, non-significant. **c** Viability of primary rat spinal cord neurons after co-culture with lentivirus-infected microglia for 24 h. *n* = 6 in 2 independent experiments. *p < 0.05; **p < 0.01; ***p < 0.001

Previous research indicates that activated microglia induce neuronal death [22–25]. To test if LRCH1 is crucial for this process, we co-cultured unprimed or primed microglia with the N27 rat dopaminergic neural cell line in Transwell 96-well plates for 24 h. Microglia and N27 cells were separated by the Transwell insert so they did not contact each other directly. As indicated in Fig. 4, primed microglia substantially induced N27 cell apoptosis and necrosis, and primed LL-infected microglia induced more apoptosis and necrosis of N27 cells as compared with primed LC-infected microglia.

To further check the effect of LRCH1, we cultured primary rat spinal cord neurons in vitro (Supplementary figure 3). We labeled these neurons with CFSE and seeded them in a Transwell 96-well plate. After that, unprimed or primed microglia were seeded in the inserts of the Transwell plate. Neurons and microglia were then co-cultured in the Transwell plate for 24 h. Neurons were then rinsed with PBS, and CFSE intensity was read on a plate reader to assess the neuron viability. As indicated in Fig. 4c, primed LL-infected microglia were more toxic to spinal cord neurons in comparison with primed LC-infected microglia.

### LRCH1 knockdown promotes the activation of p38 MAPK and Erk1/2

**Fig. 5:**
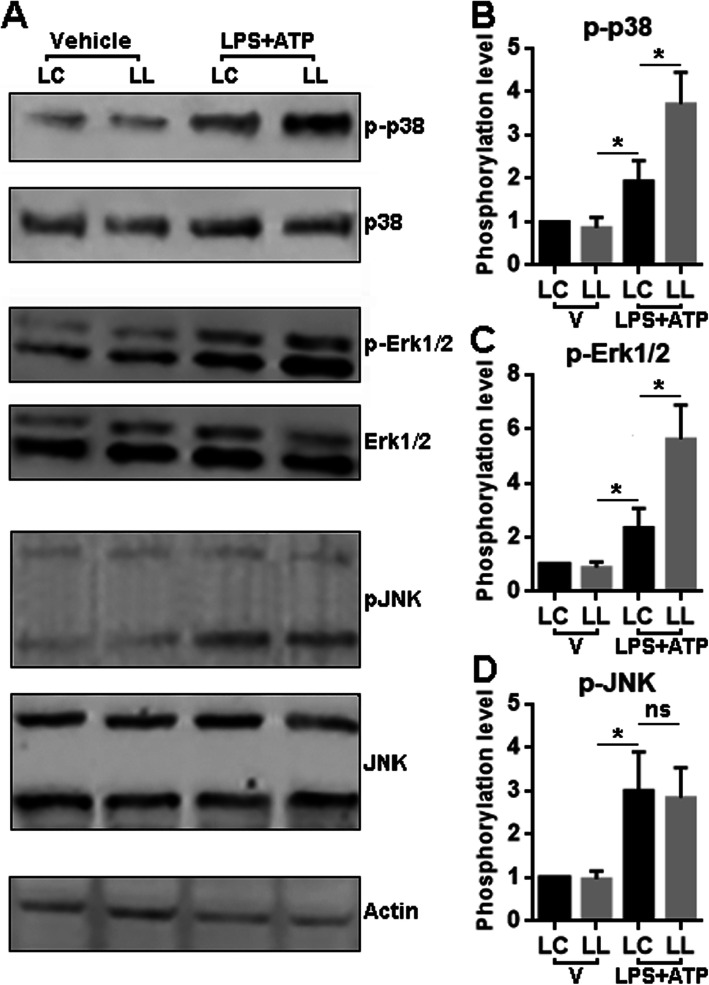
LRCH1 knockdown promotes priming-induced activation of p38 MAPK and JNK signaling. **a** Representative Immunoblotting images showing the phosphorylation of p-38 MAPK (30 min after priming), Erk1/2 phosphorylation (15 min after priming), and JNK (30 min after priming) in microglia. **b** Statistics for the phosphorylation levels (phosphorylated protein of interest: total protein of interest) of each signaling molecule in microglia with or without priming. LC, microglia infected with control lentivirus. LL, microglia infected with LRCH1 shRNA-encoding lentivirus. *p < 0.05

To find out the signal pathways that are responsible for the effect of LRCH1, we analyzed the MAPK pathways because they are critical to LPS-mediated pro-inflammatory activation of microglia [26, 27]. As shown in Fig. 5 and Supplementary figure 4, under unprimed condition, the activating phosphorylation of p38 MAPK, Erk1/2, and JNK was comparable in LC-infected microglia and LL-infected microglia. However, after priming, we observed higher phosphorylation of p38 MAPK and Erk1/2 in LL-infected microglia as compared with LC-infected microglia. The JNK phosphorylation showed no significant difference in LC-infected microglia and LL-infected microglia after priming.

### LRCH1 knockdown in microglia increases post-SCI leukocyte recruitment in vivo

**Fig. 6:**
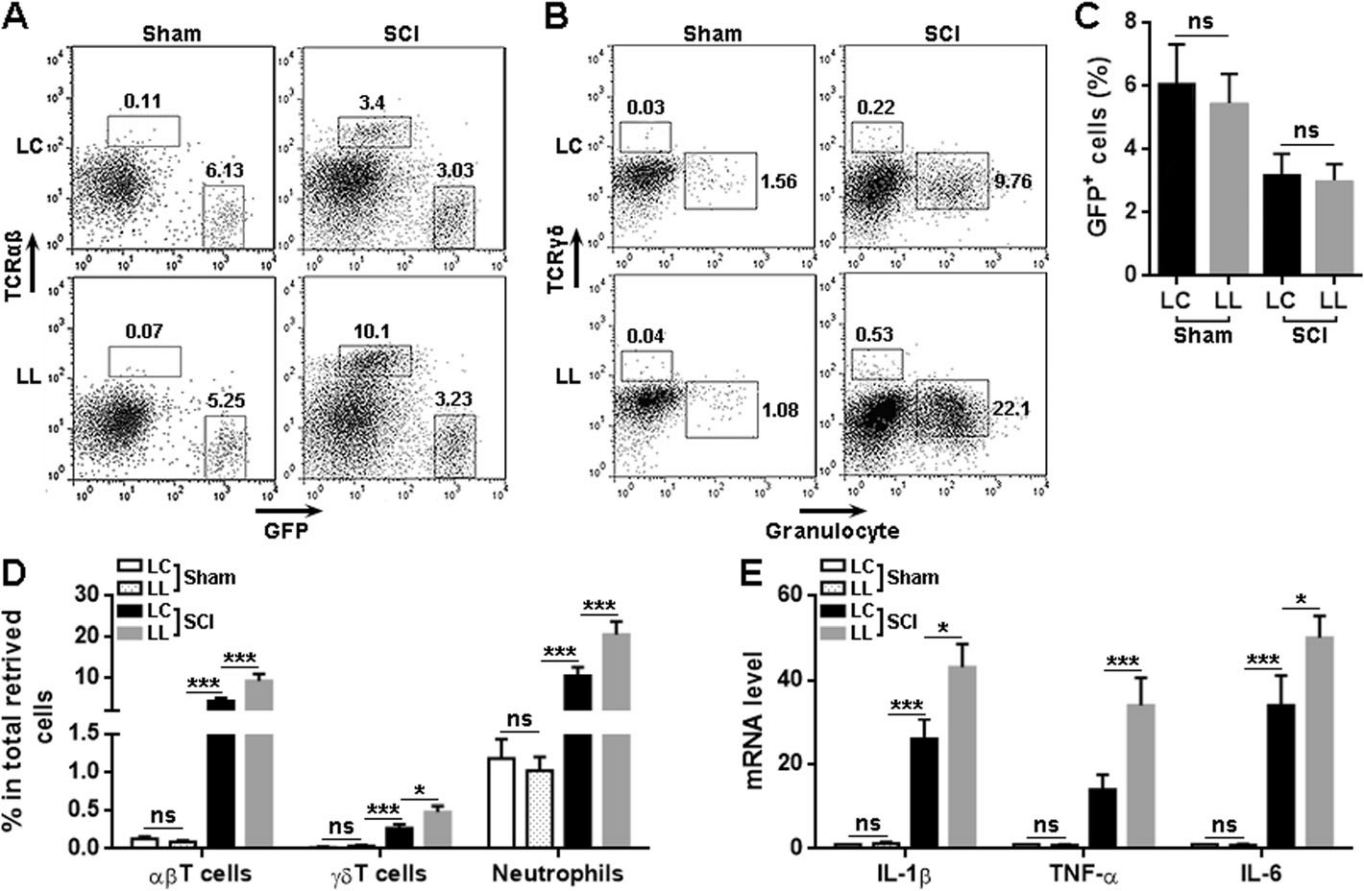
LRCH1 knockdown enhances microglia-mediated neuroinflammation after SCI. **a**, **b** Representative dot plots showing the gating of αβT cells, GFP^+^ exogenous microglia (on day 6 after SCI), as well as γδT cells and neutrophils (on day 2 after SCI) in isolated spinal cord cells. LC, rats receiving microglia infected with control lentivirus. LL, rats receiving microglia infected with LRCH1 shRNA-encoding lentivirus. **c**, **d** Statistics for the frequencies of GFP^+^ microglia (**c**) and indicated leukocyte populations (**d**) in spinal cord cells. **e** mRNA levels of indicated cytokines in GFP^+^ microglia sorted from recipient spinal cords. *n* = 5 in 3 independent experiments. ns, non-significant. *p < 0.05; **p < 0.01; ***p < 0.001

To elucidate the effect of LRCH1 in vivo, we microinjected LC-infected microglia or LL-infected microglia into the T12 spinal cord of each normal rat before SCI was conducted on these recipient rats. Staining of GFP and Iba1 indicated the presence of transferred microglia in the lesion area on day 7 after SCI (Supplementary figure 5). In addition, at indicated time points after SCI, the T12 spinal cords were taken and the injected microglia and infiltrating leukocytes were isolated and analyzed by flow cytometry. As indicated in Fig. 6 a and c, on day 3 after SCI, GFP^+^ transferred microglia were present in the spinal cords, and the frequencies of LC-infected microglia and LL-infected microglia were comparable in the recipient spinal cords. After SCI, in the spinal cords injected with LL-infected microglia, there were more infiltrating TCR_αβ_^+^ αβT cells, TCR_γδ_^+^ γδT cells, and Granulocyte^+^ neutrophils as compared with the spinal cords injected with LC-infected microglia (Fig. 6 a, b, and d). Furthermore, we retrieved the transferred microglia from the recipient spinal cords and evaluated the expression of pro-inflammatory cytokines. We found that after SCI, LL-infected microglia expressed higher IL-1β, TNF-α, and IL-6 than LC-infected microglia in the spinal cords (Fig. 6e). To check if microinjection of exogenous microglia would induce a strong inflammatory response in the spinal cords, saline or LC-infected microglia were microinjected into the spinal cords of sham-operated mice. We found that injection of microglia slightly increased the frequencies of αβT cells, γδT cells, and neutrophils, as compared with saline injection (Supplementary figure 6A to 6C). The mRNA levels of IL-1β and TNF-α were also mildly increased in the spinal cords after microglia injection (Supplementary figure 6D). Hence, the microinjection of exogenous microglia only induced very low neuroinflammation.

### LRCH1 knockdown in microglia aggravated spinal cord damage and function

**Fig. 7:**
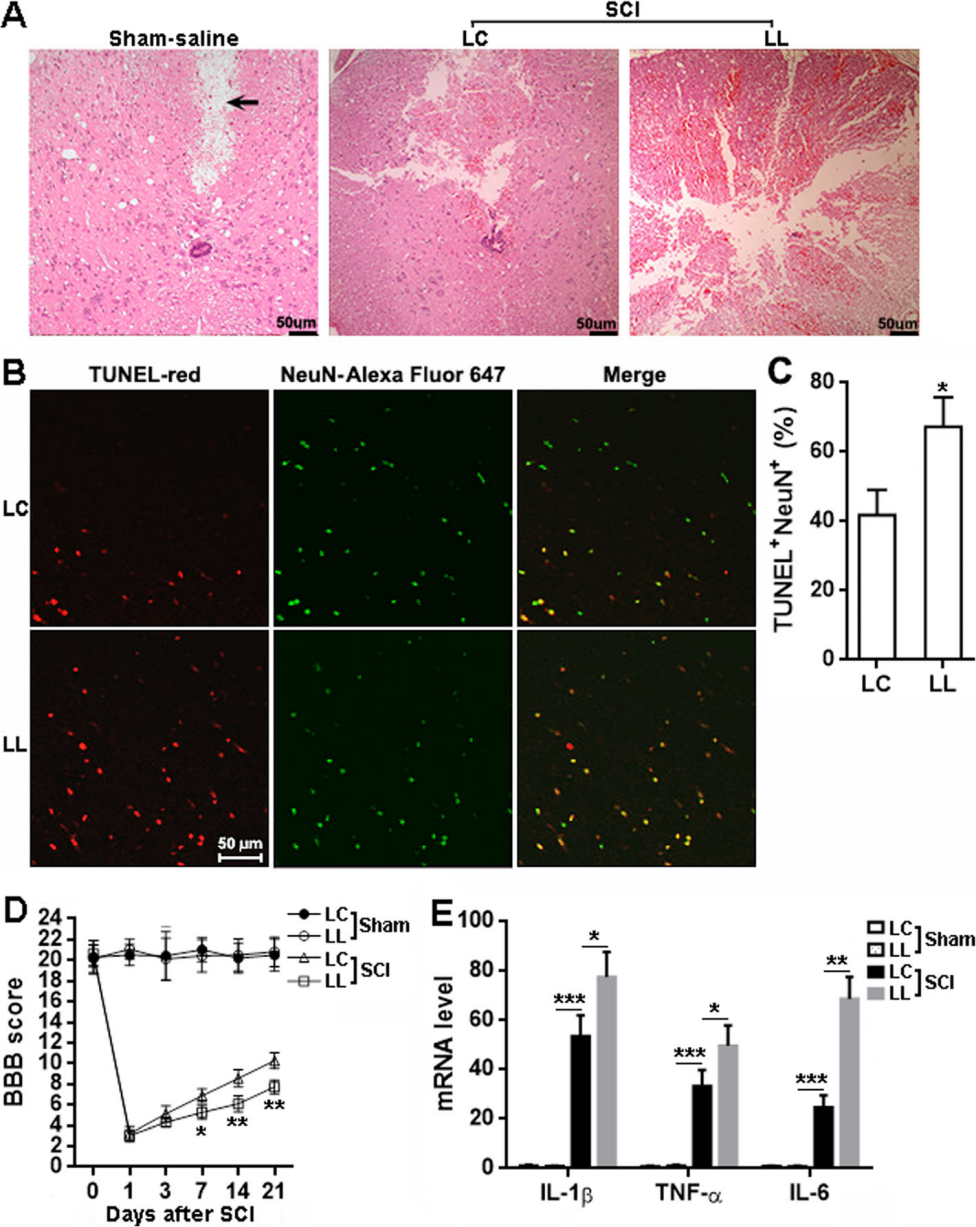
LRCH1 knockdown worsens tissue damage and locomotor function after SCI. **a** H&E staining of spinal cords on day 7 after SCI. Sham-saline, sham-operated rats injected with saline. LC, rats receiving LC-infected microglia. LL, rats receiving LL-infected microglia. The arrow indicates the tissue deficit probably caused by saline injection. **b**, **c** Double staining of TUNEL and NeuN in T12 on day 7 after SCI. Representative fluorescent images are shown in **b**. Please note that the green color of NeuN is a pseudocolor added by the imaging software, because Alexa Fluor 647 is a far-red fluorescent dye invisible to the eyes. Statistics for the proportions of TUNEL^+^NeuN^+^ cells in NeuN^+^ cells was shown in **c**. *n* = 3 in 3 independent experiments. **d** BBB scores. *n* = 8. **e** mRNA levels of indicated cytokines in T12 on day 7 after SCI. *n* = 6 in 3 independent experiments. *p < 0.05; **p < 0.01; ***p < 0.001

H&E staining displayed more extensive and severe tissue damage in the spinal cords injected with LL-infected microglia, as compared with the spinal cords injected with LC-infected microglia (Fig. 7a). Injection with saline caused mild damage to the spinal cords but did not trigger considerable inflammatory cell infiltration (Fig. 7a). After SCI, the spinal cords injected with LL-infected microglia also had more TUNEL^+^NeuN^+^ cells, i.e., apoptotic neurons, than the spinal cords injected with LC-infected microglia (Fig. 7 b and c). Moreover, the rats receiving LL-infected microglia showed a lower BBB score than the rats receiving LC-infected microglia from day 7 to day 21 after SCI, suggesting that LRCH1 knockdown aggravated post-SCI locomotor function impairment (Fig. 7d). Furthermore, the overall levels of IL-1β, TNF-α, and IL-6 were remarkably higher in the T12 spinal cord injected with LL-infected microglia, in comparison to those in the T12 spinal cord injected with LC-infected microglia (Fig. 7e).

## Discussion

Microglia are important effector cells in the injured spinal cord tissue. In the secondary phase of SCI, inflammation is the most important mechanism that directly or indirectly controls the sequelae after SCI. It is reported that microglia turn into pro-inflammatory state shortly after SCI and secrete pro-inflammatory cytokines and chemokines which mediate secondary damage following mechanical injury [28]. However, depending on the spinal cord microenvironment, microglia can also be protective after SCI, through polarization towards M2 type (alternative activation) to secrete anti-inflammatory cytokines and chemokines lead to the suppression of excessive inflammatory responses [5]. Therefore, unveiling the mechanisms by which microglial polarization is regulated is important for understanding the pathogenesis of SCI and for developing therapeutic interventions against post-SCI secondary damage.

Our study discloses for the first time the effect of LRCH1 on microglia function. We paid our attention to this molecule because our unpublished microarray data suggest a profound change in the expression of LRCH1 in microglia in the acute phase of SCI. The qRT-PCR and immunoblotting analysis confirmed the downregulation of LRCH1 in post-SCI microglia. However, the exact factors that cause this change are still unknown. To our knowledge, no previous research has suggested any clues of the transcription factor responsible for the expression of LRCH1. Our lab is using some transcription factor binding sites prediction software to analyze the potential transcription factors that bind to the promoter sequence of *Lrch1* gene locus.

According to the Human Protein Atlas, LRCH1 mRNA is present in almost every organ and tissue including the spinal cord, with the highest expression in the cerebellum (https://www.proteinatlas.org/ENSG00000136141-LRCH1/tissue). The universal expression of LRCH1 suggests its critical role in the functionality of almost all cells. It is therefore surprising that researchers have neglected this molecule for so long. Although the present study focuses on the effect of LRCH1 in microglia, it will be valuable to investigate the expression and functions of LRCH1 in neurons, astrocytes, oligodendrocytes, and macrophages in the spinal cord. Our ongoing studies are using different approaches such as imunohistochemistry, single-cell RNA sequencing, and genetic modification to ascertain the expression pattern of LRCH1 in these cell types under normal or SCI condition.

Our study demonstrates that LRCH1 might be an inhibitory factor of microglia-mediated inflammation both in vitro and in vivo. Interestingly, inhibition of LRCH1 enhanced the activation of LPS-induced p38 MAPK and Erk1/2 pathway while not interfering with the JNK pathway. p38 MAPK, Erk1/2, and JNK signaling are all critical for LSP-induced macrophage or microglial inflammatory response [29–31]. Especially, MAPKs activate activator protein 1 (AP-1) signaling [32, 33], and AP-1 supports the M1 polarization of macrophages and microglia [29, 34]. Hence, LRCH1 might restrain the microglial M1 polarization via deactivating the MAPK-AP-1 signaling. It has been reported that LRCH1 is the negative regulator of Cdc42 [11], and Cdc42 can induce the activation of p38 MAPK [35, 36]. It is, therefore, possible that inhibition of LRCH1 promotes the p38 MAPK signaling through activating Cdc42 in microglia. However, Cdc42 is considered a negative regulator of Erk1/2 [37, 38]. The effect of LRCH1 on Cdc42 could lead to the activation of Erk1/2, which was not the case in our study. Hence, the exact molecular mechanism underlying the effect of LRCH1 on MAPKs remains undetermined. In addition, LPS also activates the NF-κB pathway which plays a significant role in M1 polarization [39–41]. Interestingly, Cdc42 is reported to induce the activation of NF-κB [42, 43]. LRCH1-induced Cdc42 inhibition might also block the activation of NF-κB and subsequently regulates the production of pro-inflammatory cytokines. It will be valuable to investigate if this hypothesis is true in the future. To our knowledge, we are the first to unveil the anti-inflammatory effect of LRCH1 in microglia. A previous study indicates that LRCH1 is upregulated in foam cells by nearly 3-folds in a murine atherosclerosis model, suggesting that perhaps LRCH1 also influence macrophage polarization and function, since foam cells are fat-laden M2 macrophages [44].

It was recently reported that LRCH1 competes with Cdc42 for interaction with DOCK8 and restrains T cell migration in experimental autoimmune encephalomyelitis [11]. It is also reported that DOCK8 modulates macrophage migration through Cdc42 activation [45]. Hence, it is very likely that LRCH1 regulates microglia migration or motility under normal or pathological conditions. It will be tempting to investigate the role of LRCH1 in microglia migration, phagocytosis, and secretion of other neurotoxic or neuroprotective mediators. Meanwhile, we will continue to explore the effect of LRCH1 in the M2 polarization of microglia in the chronic stage of SCI to elucidate the significance of LRCH1 for the resolution of neuroinflammation and tissue recovery after SCI. Moreover, Cdc42 has been shown to activate MAPK signaling in certain cell types [46, 47], so perhaps Cdc42 exerts the same effect on MAPK activation in microglia, and LRCH1 blocks Cdc42 activation and subsequently inhibits MAPK activation. Our future studies will test this hypothesis.

The in vivo experiments suggest that inhibition of LRCH1 accelerates microglia-mediated neuroinflammation after SCI. So, if there is an approach, either through chemical compounds or genetic modification, to increase LRCH1 expression in microglia, it would ameliorate the outcome of post-SCI neuroinflammation and facilitate functional recovery.

## Conclusion

Our study demonstrates that inhibition of LRCH1 increases the production of pro-inflammatory cytokines from activated microglia. In addition, inhibition of LRCH1 promotes microglial polarization towards the pro-inflammatory state. The effect of LRCH1 is mediated through alleviating the activation of p38 MAPK and Erk1/2 signaling. Adoptive transfer of LRCH1-knockdown microglia worsens spinal cord injury-induced tissue damage and functional impairment. Therefore, inhibition of LRCH1 accelerates microglia-mediated neuroinflammation after spinal cord injury.

## Supplementary information

**Additional files 1: Supplementary Table 1**. Quantitative RT-PCR primers. Supplementary figure 1. GFP expression in microglia after infection lentiviral infection and puromycin selection. **Supplementary figure 2.** The mRNA levels of indicated molecules in microglia after infection with the control virus (LC). **Supplementary figure 3**. Identification of primary rat spinal cord neurons. **Supplementary figure 4**. Immunoblot membranes showing the bands of indicated molecules in LC-infected or LL-infected microglia after stimulation with LPS+ATP. **Supplementary figure 5**. Transferred microglia in injured spinal cords. **Supplementary figure 6.** Microinjection of LC-infected microglia only slightly induced inflammation in spinal cords.

## Data Availability

The datasets used and/or analyzed during the current study are available from the corresponding author on reasonable request.

## Abbreviations

SCI: Spinal cord injury
LRCH1: Leucine-rich repeats and calponin homology domain containing 1
DOCK8: Dedicator of cytokinesis 8
PBS: Phosphate-buffered saline
FBS: Fetal bovine serum
iNOS: Inducible nitric oxide synthase
MAPK: Mitogen-activated protein kinase
JNK: Jun N-terminal kinase
TUNEL: Terminal deoxynucleotidyl transferase dUTP nick end labeling
AP-1: Activator protein 1
