# Supplementary material for: Inhibition of leucine-rich repeats and calponin homology domain containing 1 accelerates microglia-mediated neuroinflammation in a rat traumatic spinal cord injury model
Source: J Neuroinflammation. 2020 Jul 6;17:202. doi: 10.1186/s12974-020-01884-4 (PMC7339506; doi:10.1186/s12974-020-01884-4)
Supplement: Supplementary file 1 — Additional files 1: Supplementary Table 1. Quantitative RT-PCR primers. Supplementary figure 1. GFP expression in microglia after infection lentiviral infection and puromycin selection. Supplementary figure 2. The mRNA levels of indicated molecules in microglia after infection with the control virus (LC). Supplementary figure 3. Identification of primary rat spinal cord neurons. Supplementary figure 4. Immunoblot membranes showing the bands of indicated molecules in LC-infected or LL-infected microglia after stimulation with LPS+ATP. Supplementary figure 5. Transferred microglia in injured spinal cords. Supplementary figure 6. Microinjection of LC-infected microglia only slightly induced inflammation in spinal cords. [file 12974_2020_1884_MOESM1_ESM.docx]

**Supplementary information**

| **Molecule** | **Forward (5’ to 3’)** | **Reverse (5’ to 3’)** |
| --- | --- | --- |
| LRCH1 | TGGCAGCTCAGGACAAGAAA | TCCGTGGTTAGAAGTTCTTCCG |
| IL-1β | TGTCTGACCCATGTGAGCTG | GCCACAGGGATTTTGTCGTT |
| TNF-α | TCGGTCCCAACAAGGAGGAG | GGGCTTGTCACTCGAGTTTTG |
| IL-6 | ACTTCACAAGTCGGAGGCTT | TTCTGACAGTGCATCATCGCT |
| β-actin | ACAACCTTCTTGCAGCTCCTC | CTGACCCATACCCACCATCAC |

**Supplementary Table 1. Quantitative RT-PCR primers**

**
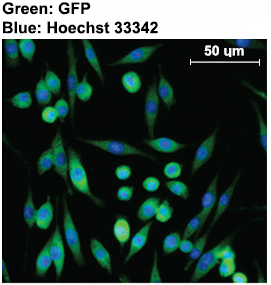
**

# Supplementary figure 1. GFP expression in microglia after infection lentiviral infection and puromycin selection. Primary rat spinal cord microglia were infected with LC and selected with puromycin as described in Materials and Methods. Microglia were then incubated in PBS containing 1 µg/ml Hoechst 33342 at room temperature for 15 minutes, followed by observance on a Zeiss Axiovert 200 fluorescence microscope. Original magnification = 200×.

**
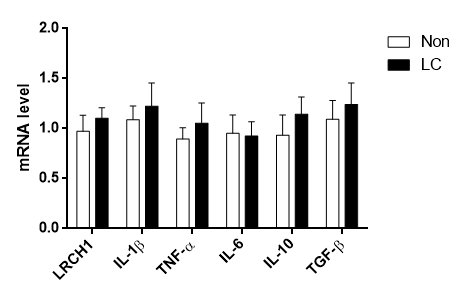
**

**Supplementary figure 2. The mRNA levels of indicated molecules in microglia after infection with the control virus (LC).** Primary microglia were infected with LC particles at the MOI of 20 and incubated overnight. The next morning the medium was then replaced with fresh medium. Cells were incubated in fresh medium for 2 days, followed by incubation with 2 μg/ml puromycin (Sigma-Aldrich) for 4 days. Non: non-infected microglia. LC: LC-infected microglia.


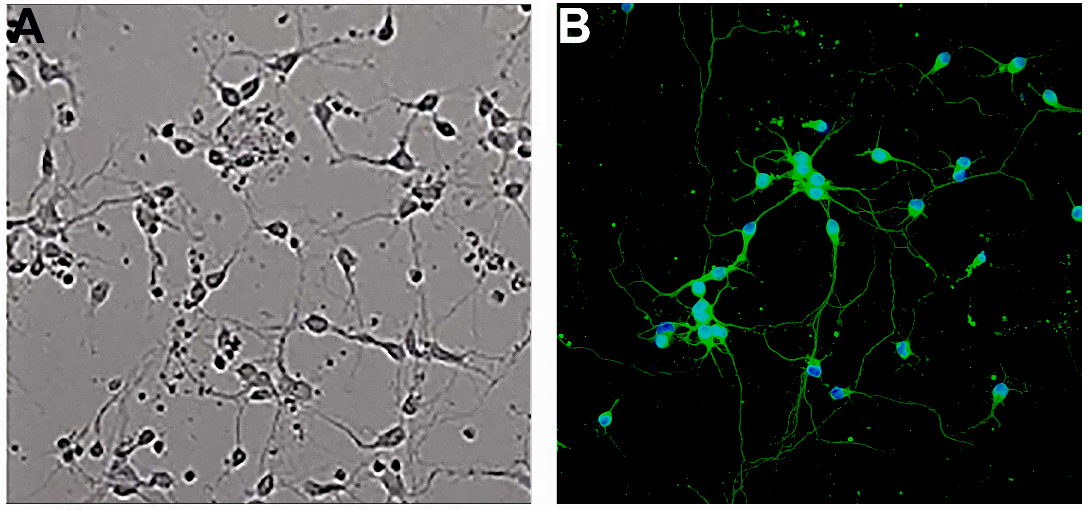


**Supplementary figure 3. Identification of primary rat spinal cord neurons.** The primary rat spinal cord neurons were purchased from Guangzhou Ubigene Biosciences. **(A)** The neurons cultured in the Transwell plate were shown in the light field. **(B)** Immunofluorescent staining of neurons with 5 µg/ml MAP2 antibody. Original magnification = 100 ×.





**Supplementary figure 4. Immunoblot membranes showing the bands of indicated molecules in LC-infected or LL-infected microglia after stimulation with LPS+ATP**. LC: LC-infected microglia. LL: LL-infected microglia.


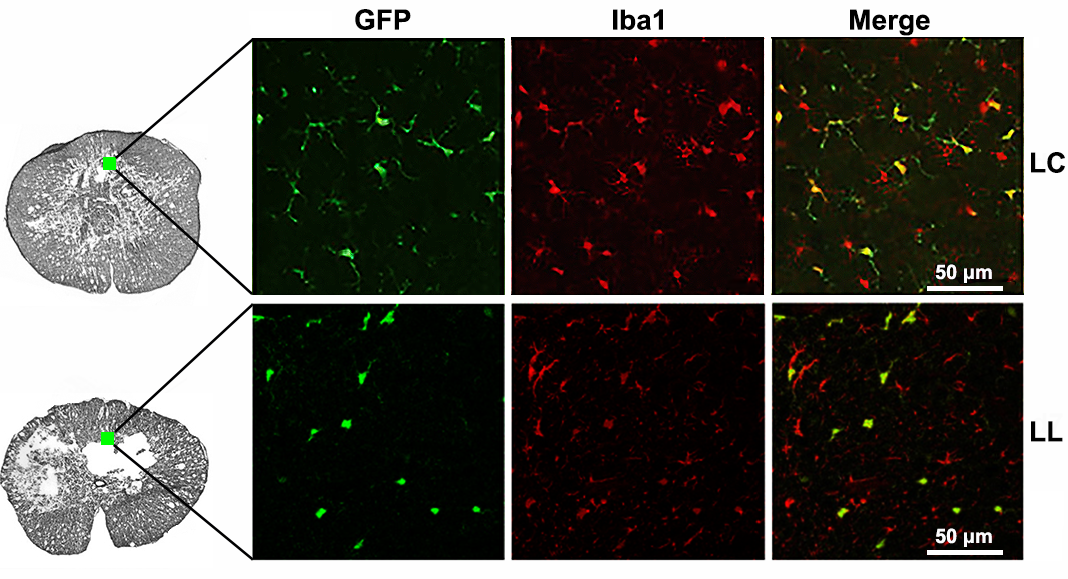


**Supplementary figure 5. Transferred microglia in injured spinal cords.** Lentivirus-infected microglia were injected into rat spinal cords immediately before SCI. Staining of GFP and Iba1 in coronal sections of spinal cords on day 7 after SCI. Green square: the selected field.


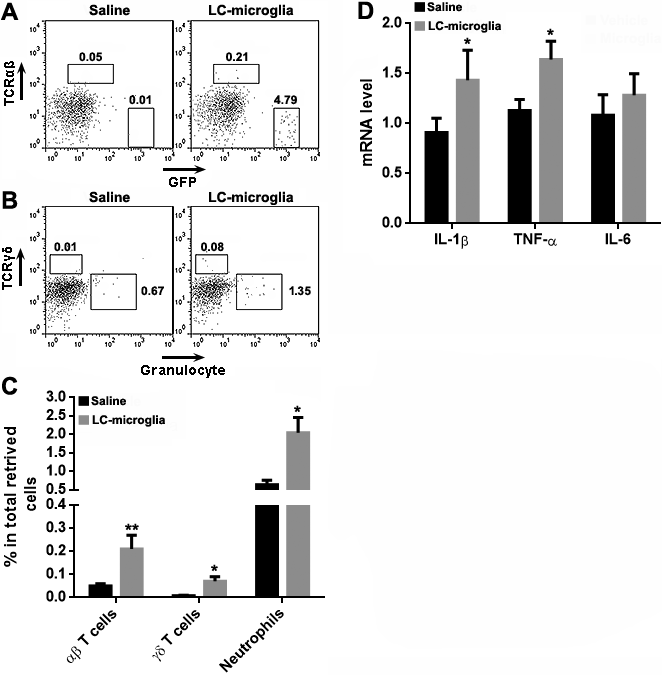


**Supplementary figure 6. Microinjection of LC-infected microglia only slightly induced inflammation in spinal cords.** Sham-operated mice received microinjection of either saline or LC-infected microglia as described in Materials and Methods. **(A and B)** Representative dot plots showing the gating of αβT cells and GFP^+^ exogenous microglia (on day 6 after injection), as well as γδT cells and neutrophils (on day 2 after injection) in isolated spinal cord cells. Saline: Saline injection. LC-microglia: Injection with LC-infected microglia. **(C)** Statistics for the frequencies of indicated leukocyte populations in recovered spinal cord cells. **(D)** mRNA levels of indicated cytokines in T12 on day 6 after injection. n=3 per group. *, p<0.05; **, p<0.01.
